# Multiple electrolyte disorders triggered by proton pump inhibitor-induced hypomagnesemia: Case reports with a mini-review of the literature 

**DOI:** 10.5414/CNCS111284

**Published:** 2024-01-04

**Authors:** Camila Costa Souza, Larissa G. Rigueto, Henrique Costa Santiago, Antonio Carlos Seguro, Adriana Castello Girardi, Weverton Machado Luchi

**Affiliations:** 1 Hospital Universitário Cassiano Antonio Moraes, Universidade Federal do Espírito Santo (HUCAM-UFES), Vitória, ES,; 2 Laboratorio de Investigacao Medica 12 (LIM12), and; 3 Laboratorio de Genetica e Cardiologia Molecular, Instituto do Coracao (InCor), Hospital das Clinicas HCFMUSP, Faculdade de Medicina, Universidade de Sao Paulo, Sao Paulo, SP, Brasil; *Contributed equally to this work

**Keywords:** hypomagnesemia, proton pump inhibitors, electrolyte disorders, omeprazole, case report

## Abstract

Drug-induced hypomagnesemia is an adverse effect with the potential for serious and fatal outcomes. Although rare, chronic use of proton pump inhibitors (PPIs) can cause hypomagnesemia due to impaired intestinal absorption, mainly attributed to reduced transcellular transport of magnesium via transient receptor potential melastatin 6 (TRPM6) and 7 (TRPM7) channels. However, a reduction of magnesium paracellular absorption due to the downregulation of intestinal claudins has also been reported. PPI-induced hypomagnesemia can trigger other concomitant electrolyte derangements, including hypokalemia, hypocalcemia, hypophosphatemia, and hyponatremia. Here we report two cases of multiple electrolyte disorders associated with PPI-induced hypomagnesemia, the clinical manifestations of which were cardiac arrhythmia, cognitive changes, and seizure crisis. These cases illustrate the need to monitor serum magnesium levels in patients on long-term PPI use, especially in the elderly and those with malabsorptive bowel syndromes or taking loop diuretics and thiazides.

## Introduction 

Proton pump inhibitors (PPIs) are widely prescribed for managing peptic ulcers and gastroesophageal reflux disease (GERD). Their action in reducing gastric acid secretion effectively prevents dyspeptic symptoms and alleviates undesired effects of other medications, particularly nonsteroidal anti-inflammatory drugs. The widespread use of PPIs has allowed for adverse effects to be more frequently reported, particularly with prolonged use, contradicting the premise of their absolute harmlessness [1]. 

Increasing evidence associates PPIs with adverse renal, cardiovascular, bone, pulmonary, and neurological effects [1]. Prolonged use is associated with electrolyte disorders, with a highlight on hypomagnesemia [2]. The main mechanism described for PPI-induced hypomagnesemia involves the inhibition of intestinal magnesium absorption through melastatin-type transient receptor potential (TRPM) channels 6 and 7 [3]. However, a reduction of magnesium paracellular absorption due to the downregulation of intestinal claudins has also been reported [4]. 

Severe hypomagnesemia can trigger cardiac arrhythmias, seizures, and multiple secondary electrolyte disorders, including hypokalemia, hypocalcemia, and hyponatremia [3]. Here, we report two cases of severe hypomagnesemia secondary to prolonged omeprazole therapy. With the increased number of prescriptions and indiscriminate use of these drugs, we emphasize the importance of monitoring serum magnesium levels in patients under chronic use of PPIs. 

## Case reports 

The CARE Checklist has been completed by the authors for this case report and is attached as Supplemental Material. 

### Patient 1 

A male patient, 35 years old, obese, hypertensive, with GERD, and using omeprazole and hydrochlorothiazide for 5 years complained of nonspecific retrosternal pain without relation with physical effort or aggravating factors, which had started 4 days before seeking emergency care. He also reported paresthesia and muscle stiffness in the face, and cramps in the upper and lower limbs. The physical exam revealed the presence of the Trousseau sign (carpal spasm) without other alterations. The electrocardiogram showed a prolonged QT interval (507 ms) and no signs of ischemia. Laboratory tests showed negative markers of myocardial necrosis and reduced levels of magnesium (Mg^2+^ = 0.8 mg/dL), potassium (K^+^ = 2.6 mEq/L), and ionic calcium (Ca_i_^2+^ = 3.2 mg/dL), associated with a reduced urinary excretion fraction (FE) of Mg^2+^ (FEMg^2+^) (Table 1). Initially, the etiology of electrolytic disturbances was attributed to the use of hydrochlorothiazide. However, a reduced FEMg^2+^ associated with hypocalcemia ruled out the initial suspicion, suggesting the diagnosis of extrarenal hypomagnesemia related to chronic use of PPI, with secondary hypokalemia and hypocalcemia. The patient showed clinical improvement after intravenous electrolytic replacement, with a marked transitory elevation of parathyroid hormone (PTH = 407 pg/mL) levels on the 2^nd^ day of Mg^2+^ replacement, which reduced as serum magnesium levels normalized during hospitalization (Table 1). Despite multiple advises, the patient kept using omeprazole after discharge. On follow-up, he had a new episode of hypomagnesemia (1.5 mg/dL), even with oral Mg^2+^ replacement and spironolactone. Later, PPI was definitively discontinued, and during 1 year of follow-up, serum electrolytic levels remained normal (Table 1). 

### Patient 2 

A 65-year-old female patient with generalized anxiety disorder, hypertension, and a history of hypokalemia over the last 2 years was taking valsartan, amlodipine, chlorthalidone, diazepam, and omeprazole. Omeprazole was initiated 5 years before to manage GERD. She was admitted because of muscle weakness, tremors in the upper limbs, nausea, and intense headache, which evolved into two generalized tonic-clonic seizures. She also reported that previously she was being assisted by a neurologist for Parkinson’s disease investigation due to tremors and cognitive decline in the last month. Admission tests revealed hypomagnesemia (0.6 mg/dL), hypokalemia (2.7 mEq/L), and hypocalcemia (3.15 mg/dL), with normal cranial MRI. The clinical picture was therefore attributed to electrolytic disorders, prompting the initiation of intravenous electrolyte correction. Additionally, the prescription of thiazide diuretics was suspended. On the 3^rd^ day of Mg^2+^ replacement, the patient displayed a FEMg^2+^ of 12.4%. However, on the 5^th^ day after the suspension of magnesium infusion, there was a recurrence of hypomagnesemia (0.7 mg/dL), and the FEMg^2+^ at this time, before the start of the new replacement, was 0.5%, thereby ruling out that the origin of the hypomagnesemia was renal magnesium wasting. Thus, the main diagnostic hypothesis became hypomagnesemia induced by chronic use of omeprazole. Indeed, the patient improved progressively after suspension of the PPI, maintaining normal levels of Mg^2+^ and other electrolytes (Table 1). In addition, her cognitive level improved, and her tremor symptoms stopped. 

## Discussion 

PPIs irreversibly inhibit the H^+^/K^+^-ATPase pump, preventing the final stage of acid secretion by parietal cells in the stomach. These drugs are considered safe and, in most countries, sold without a medical prescription. However, recent publications question its harmlessness, as adverse effects are being increasingly reported. Prolonged therapy has been associated with increased *Clostridium difficile* infections, osteopenia and fractures, renal parenchymal disease, electrolytic disorders, cutaneous lupus, acute myocardial infarction, dementia, and hospital-acquired pneumonia. The main risk factor for adverse manifestations is prolonged use which can be potentiated by high doses [1, 5]. 

Hypomagnesemia is the electrolyte imbalance that raises the greatest concern when prescribing PPIs [2]. A recent meta-analysis of 16 observational studies, including 131,507 patients, reported a 71% increase in the risk of hypomagnesemia in patients receiving PPIs, suggesting a causal association [6]. In 2011, the FDA issued a warning about the possibility of hypomagnesemia secondary to PPI use and recommended monitoring serum Mg^2+^ levels in chronic users, especially in those with additional risk factors, such as older adults using Mg^2+^-depleting diuretics (loop and thiazide diuretics), and those with gastrointestinal absorption disorders [5]. 

**Figure 1.**
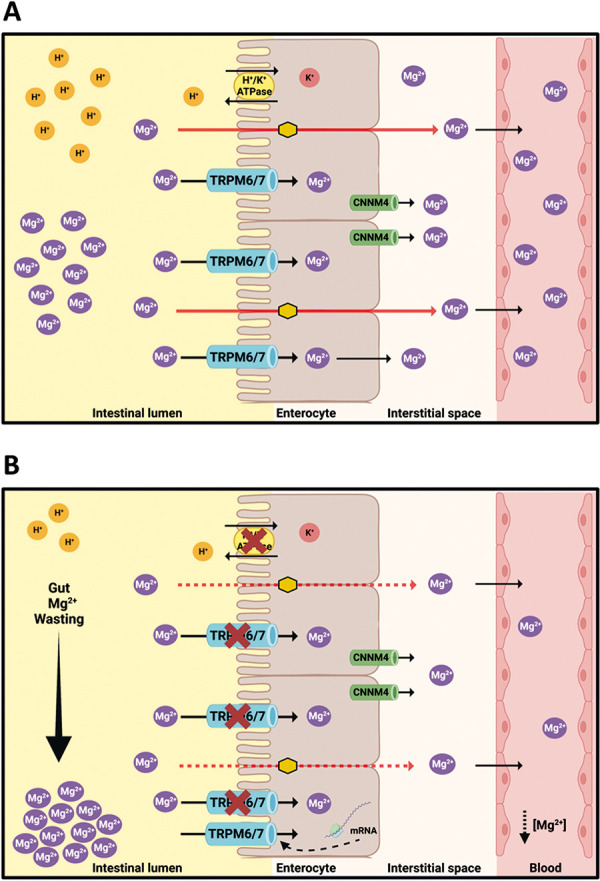
Influence of proton pump inhibitors (PPIs) on intestinal absorption of Mg2+. A: Mg^2+^ is absorbed into the enterocytes via the paracellular (solid red lines) and the transcellular route (through TRPM6/7) (solid black lines). After absorption, it enters the interstitial space via CNNM4 and, finally, the bloodstream via the portal vein. B: PPIs inhibit Mg^2+^ absorption by increasing the intestinal lumen pH, both by gastric and colonic (non-gastric) H^+^/K^+^ATPase antagonism. In this condition, the affinity of TRPM6/7 for Mg^2+^ decreases, resulting in lower intestinal absorption, despite the compensatory increase in TRPM6 channel expression (dotted black curved line). Additionally, paracellular transport through claudins (orange hexagons) is compromised with increased intestinal pH (dotted red lines). TRPM = transient receptor potential channel of melastatin; CNNM4 = cyclin M4.

Recent studies show that hypomagnesemia secondary to PPIs is not due to renal loss of Mg^2+^ but decreased gastrointestinal absorption [4, 7]. It is hypothesized that the concentration of H^+^ is essential for intestinal lumen Mg^2+^ absorption through TRPM 6 and 7 channels and claudins [4]. Thus, the increase in pH induced by PPI might impair Mg^2+^ absorption (Figure 1). On the other hand, when analyzing other drugs associated with hypomagnesemia, the vast majority cause renal loss of Mg^2+^. The evaluation of FEMg^2+^ in the PPI use scenarios can be of great diagnostic help, especially when another medication can also contribute to hypomagnesemia, as in the 2 cases described above. In the presence of hypomagnesemia, a FEMg^2+^ < 2% indicates that the cause is related to extrarenal losses [8]. 

When serum magnesium is between 1.2 and 1.8 mg/dL, hypomagnesemia is often asymptomatic or has mild symptoms, such as fatigue. Symptoms usually develop with serum levels less than or equal to 1.2 mg/dL. Nevertheless, studies point out that even in patients without symptoms, there is an increased risk of atrial fibrillation, left ventricular hypertrophy, insulin resistance, and all-cause and cardiovascular mortality. On the other hand, severe hypomagnesemia (< 1 mg/dL) typically presents with tetany, seizures, bradycardia, hypotension, and death. The most feared clinical manifestations are neuromuscular (cramps or muscle weakness, ataxia, carpopedal spasm, tetany, vertigo, seizures, depression, cognitive decline, and psychosis) and cardiovascular system disease (ventricular arrhythmias – torsades de pointes and supraventricular tachycardia) [8]. 

**Figure 2.**
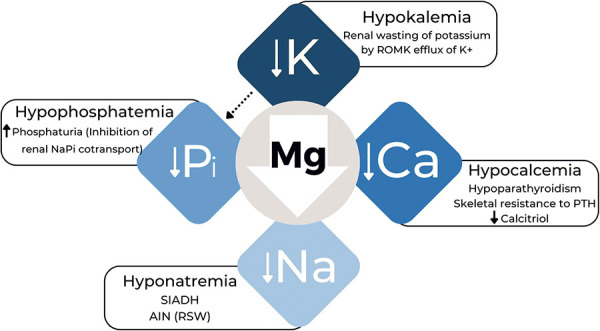
Multiple electrolyte disorders due to hypomagnesemia induced by proton pump inhibitors. Hypomagnesemia caused by the reduction of intestinal absorption provokes a series of other concomitant electrolyte disturbances, highlighted by hypokalemia and hypocalcemia and, less frequently, hypophosphatemia and hyponatremia. Hypokalemia itself can aggravate phosphaturia (black dotted arrow). ROMK = medullary renal outer potassium channel; NaPi = sodium-phosphorus cotransport present in the kidney; PTH = parathyroid hormone; SIADH = syndrome of inappropriate antidiuretic hormone secretion; AIN = acute interstitial nephritis; RSW = renal salt wasting.

Low serum levels of Mg^2+^ can induce multiple electrolyte disturbances, including hypokalemia, hypocalcemia, hypophosphatemia, and, more rarely, hyponatremia (Figure 2) [3]. Hypomagnesemia occurs between 3 months and 1 year after starting the PPIs. After discontinuing the drug, Mg^2+^ serum levels tend to normalize within ~ 1 – 2 weeks, and recurrence is frequent when these drugs are reintroduced, as observed in patient 1 (Table 1). The finding of asymptomatic hypocalcemia or hypokalemia in PPI users often indicates a Mg^2+^ deficiency disorder [2, 9]. 

Hypokalemia results from renal potassium loss through the renal medullary outer potassium channel (ROMK). A threshold of intracellular Mg^2+^ concentration is required to inhibit the opening of the ROMK channel and prevent potassium efflux from the renal tubular cell. Thus, in scenarios of hypomagnesemia and low intracellular Mg^2+^ concentration, potassium efflux occurs in the tubular lumen, resulting in kaliuresis [9]. 

Hypomagnesemia can cause functional hypoparathyroidism, which is classically described as hypocalcemia associated with a low or inappropriately normal PTH level [10]. Serum Mg^2+^ < 1.2 mg/dL can block PTH secretion by disinhibiting the alpha subunit of the G protein (Gα protein), which activates the calcium-sensing receptor (CaSR) in the parathyroids and thus impairs the hormone release. In this context, as soon as intravenous Mg^2+^ supplementation begins, PTH secretion increases within a few minutes, as observed in patient 1 (Table 1) [11]. This laboratory response is compatible with an inability to secrete PTH rather than inhibit its biosynthesis. Another contributing factor to hypocalcemia is PTH resistance to its receptor in bones and kidneys called parathyroid hormone 1 receptor (PTH1R), a G protein-coupled adenylate cyclase-dependent receptor. Since Mg^2+^ is an adenylate cyclase cofactor, its deficiency compromises PTHR1 signaling and stimulus transmission [12]. Additionally, calcitriol production via 1-α-hydroxylase is also compromised by Mg^2+^ deficiency, contributing to hypocalcemia [13]. 

Hypophosphatemia can also be associated with hypomagnesemia, although less common. Its pathogenesis involves changes in the expression and distribution of sodium-phosphate (NaPi) cotransporters located in the renal tubular epithelial cells of the proximal convoluted tubule (PCT). Ginn and Shanbur [14] demonstrated increased phosphaturia in rats subjected to a magnesium-restricted diet, establishing a link between hypomagnesemia and a reduction in the maximum rate of tubular phosphate reabsorption (TmP). Additionally, hypokalemia, which is frequent in the context of Mg^2+^ deficiency, reduces the expression of the NaPi subtype IIc transporter along the brush border in the PCT, worsening phosphaturia [15]. 

The use of PPIs is a rare cause of hyponatremia. When reported, the most frequently associated PPI is omeprazole [16]. An experimental study on the renal collecting duct of rabbits demonstrated that Mg^2+^ inhibits the action of the antidiuretic hormone [17]. It is speculated that Mg^2+^ deficiency may be related to the syndrome of inappropriate antidiuresis. Another possible mechanism would be salt-losing nephropathy within the context of acute interstitial nephritis induced by PPIs [16, 18]. In addition, Mg^2+^ acts as a cofactor of the Na^+^/K^+^ ATPase enzyme, and its inhibition could contribute to the generation of hyponatremia [19]. 

Treating hypomagnesemia secondary to PPIs is similar to hypomagnesemia of other etiologies. Parenteral Mg^2+^ replacement is indicated in severe cases, along with PPI suspension. The need to start calcium, potassium, and phosphate replacements should be evaluated simultaneously. We reinforce the importance of collecting urinary ions for analysis of FEMg^2+^ immediately before the start of electrolyte replacement to avoid misinterpretation of the results, which can occur when the analysis is carried out during venous Mg^2+^ infusion, as observed in patient 2 (Table 1). In healthy individuals, more than 80% of infused magnesium is excreted within 48 hours [20]. Thus, if Mg^2+^ replacement has already started before urine collection for FEMg^+2^ calculation, we suggest a new urine analysis after 3 – 5 days of intravenous replacement interruption. 

Even after the resolution of hypomagnesemia, it is advisable to maintain oral supplementation, preferably with magnesium oxide, for a few months to replenish the body’s stores of this electrolyte. It is of clinical relevance to emphasize that when diagnosis of PPI-induced hypomagnesemia is established, replacing the PPI with another of the same pharmacologic class does not prevent recurrence of the electrolyte disorder. In patients with gastric acid diseases who present with electrolyte disorders, replacement of PPIs with histamine H2 receptor antagonists is recommended. Regular magnesium supplementation is indicated when it is necessary to maintain the PPIs [21]. Monitoring serum Mg^2+^ levels in PPI users should be considered in the following scenarios: (i) any patient with signs or symptoms potentially associated with hypomagnesemia, hypocalcemia, hypophosphatemia, hypocalcemia, and hyponatremia; (ii) use of PPIs for 3 – 6 months or more, especially in the elderly patient, patients using loop or thiazide diuretics or those with gastrointestinal disorders that favor hypomagnesemia [5]. 

## Ethics statement 

This study protocol was reviewed and approved by the Ethics Committee of the Hospital Universitario Cassiano Antonio Moraes – HUCAM, approval number 5.983.602. Written informed consent was obtained from the patients to publish this case report and any accompanying images. 

## Data availability 

All data generated or analyzed during this study are included in this article. Further inquiries can be directed to the corresponding author. 

## Funding 

The authors received no financial support for the research, authorship, and/or publication of this article. 

## Conflict of interest 

The authors declare no potential conflicts of interest concerning this article’s research, authorship, and/or publication. 

**Table 1.** Summary of the patients’ blood and urine test results.

	Patient 1						Patient 2						Reference values
	Hospitalization			After hospital discharge			Hospitalization					After hospital discharge	
	D1	D2	D3	Omeprazole restarting		Omeprazole interruption	D1	D3	D4	D9	D15	1° Outpatient consultation	
Magnesium	0.8	2.5	2.3	1.5	1	2	0.6	1.4	1.7	0.7	2.1	2	1.8 – 2.4 mg/dL
Ionized calcium	3.2	4.1	4.1	4.6	4.1	5	3.1	–	–	–	4.9	5.2	4.4 – 5.4 mg/dL
Sodium	138	135	135	136	142	137	144	141	137	–	135	140	135 – 145 mEq/L
Potassium	2.6	3.1	3.8	4	3.2	4.6	2.7	3.4	5	5	5	4.7	3.5 – 5 mEq/L
Phosphate	2.9	2.9		3.2	3.7	4.5	–	2.2	–	–	4.3	4.1	2.5 – 4.5 mg/dL
PTH	35	407*	185	89	59	53	–	–	64	30	–	34	15 – 68 pg/mL
Creatinine	0.62	0.8	0.66	0.84	0.9	0.92	0.98	0.81	0.85	1.4	1.1	0.99	0.7 – 1.2 mg/dL
Urea	19	21	17	26	26	35	22	19	26	48	35	30	19 – 44 mg/dL
FEMg^2+^	0.25%	–	–	0.17%	0.23%	2.5%	–	12%**	–	0.5%***	–	5.4%	2 – 4%
FEK^+^	15%	–	–	–	–	4.4%	–	–	–	–	–	–	4 – 16%
TRP	82%	–	–	–	–	–	–	–	–	–	–	–	86 – 99%
TmP/TGF	2.3	–	–	–	–	–	–	–	–	–	–	–	2.6 – 3.8 mg/dL

*Patient 1: 24 hours after intravenous infusion of magnesium sulfate; **Patient 2: elevation of FEMg^2+^ during intravenous supplementation (inducing a false interpretation of renal loss); ***Patient 2: Without intravenous supplementation, characterizing extrarenal loss; TRP = (1 – phosphorus excretion fraction) × 100; TmP/GFR = [(0.3 × TRP) / (1 – (0.8 × TRP)] × serum phosphorus; FEK^+^ = (UK × PCr) / (PK × UCr)] × 100; FEMg^2+^ = (UMg × PCr) / (PMg × UCr × 0.7)] × 100. D = day; FEK^+^ = fractional excretion of potassium; FEMg^+2^ = fractional excretion of magnesium; PTH = parathyroid hormone; TRP = tubular reabsorption of phosphate; TmP/GFR = transport maximum for phosphate reabsorption/glomerular filtration rate.

## Supplemental material

Supplemental material Reporting checklist for case report or case series. Based on the CARE guidelines.
